# The Cultural Intelligence Scale (CQS): A Contribution to the Italian Validation

**DOI:** 10.3389/fpsyg.2018.01183

**Published:** 2018-07-10

**Authors:** Caterina Gozzoli, Diletta Gazzaroli

**Affiliations:** Department of Psychology, Università Cattolica del Sacro Cuore, Milan, Italy

**Keywords:** cultural intelligence, self-report measure, Italian validation, globalization, confirmatory factor analysis

## Abstract

In the current globalized working context, professionals are asked to be able to implement specific competences. Cultural Intelligence is a construct referring to an individual’s ability to function and manage effectively in culturally diverse settings and is conceived as an aggregate multidimensional construct. Purpose of this study was to examine the validity of score interpretations of the Italian version of the Cultural Intelligence Scale (CQS). CQS is aimed to measure individual ability to understand, act and manage effectively in culturally diverse settings. Participants were 755 professionals (females = 64.2%) from different organizational contexts, ranging from 20 to 63 years old (*M* = 40.4; *SD* = 10.29). Data were collected with the Italian translated version of the CQS. Results of confirmatory factor analysis (CFA) suggested good data-model fit. As proposed in the original version, CQS is composed of 20 items and four different theoretical dimensions (Metacognitive, Cognitive, Motivational, and Behavioral) that correlate with each other. This study could be considered a first contribution to fill the lack of self-report measure concerning cultural intelligence in the Italian context with a scale showing promising results.

## Introduction

Nowadays within organizational contexts the element of diversity is becoming increasingly critical and present. In many cases diversity is related to the cultural dimension because of:

- socio-demographic and political changes that distinguish the modern era;
- the progressive internationalization of work due to global production and consumption.

However, while “globalization has made the world seem smaller and “flat” in many ways (Friedman, 2005), increasing cultural diversity creates challenges for individuals and organizations (making the world “not so flat” after all). […]. Relatively little research focuses on factors that could improve intercultural encounters (Gelfand et al., 2007) […] leaving an important gap in our understanding of why some individuals are more effective than others in culturally diverse situations” (Ang et al., 2007, pp. 335–336). As Ang et al. (2007) highlighted, organizational contexts are increasingly contaminated by “elements of cultural diversity.” By the way, we deal with this argument bearing in mind that cultural diversity is not the only form of diversity, but one of the many forms that it may take (religious, generational, gender, competence, and training, etc.). Indeed, the relationship with Otherness itself implies the need to fully relate to diversity in all its forms (Gozzoli, 2016a,b).

One of the most frequent mistakes of our thinking and acting is non-recognition of diversity, assuming a tendency to judge behaviors and situations through our own perspective, considering it to be universally shared (ethnocentrism). Most of the time we do not pay specific attention to the bond between the culture we are surrounded by and our own values, certainties, opinions. Culture is everything concerning our “way of living.” We come into the world and grow up immersed in a specific culture that we assimilate unconsciously every day. In other words, our own culture is so assumed and weighty that it becomes invisible. Of course, this attitude–even if unconscious in most cases–may lead to communicative and relational conflicts, misunderstandings and inefficiency.

Translated into professional contexts, it means being able to develop specific skills. Therefore, developing an intercultural competence could allow organizations being more open to diversity and innovation.

In the Italian context there’s not yet adequate reflection about this issue, that’s the reason why the Cultural Intelligence construct by Earley and Ang (2003) seems pertinent. “Cultural Intelligence (CQ), defined as an individual’s capability to function and manage effectively in culturally diverse settings, is consistent with Schmidt and Hunter’s (2000, p. 3) definition of general intelligence as ‘the ability to grasp and reason correctly with abstractions (concepts) and solve problem’s” (Ang et al., 2007, p. 337).

Cultural Intelligence is conceived as an aggregate multidimensional construct. In line with Sternberg’s (1986) multiple-loci of intelligence theory, the authors propose four CQ dimensions: metacognitive, cognitive, motivational, and behavioral. Metacognitive CQ reflects higher-order cognitive processes used to acquire and understand cultural knowledge (Flavell, 1979). Relevant capabilities include planning, monitoring, and revising mental models of cultural norms for countries or groups of people. Those with a high Metacognitive CQ (Brislin et al., 2006; Triandis, 2006) are consciously aware of others’ cultural preferences before and during interactions, question cultural assumptions and adjust their mental models during and after interactions. Cognitive CQ reflects knowledge of the norms, practices, conventions, economic, legal and social systems, and knowledge of basic frameworks of values in different cultures acquired through education and personal experiences (Triandis, 1994; Hofstede, 2001). Those with high Cognitive CQ understand similarities and differences across cultures (Brislin et al., 2006). Motivational CQ reflects the capability to direct attention and energy toward learning about and functioning in situations distinguished by cultural differences. Those with high Motivational CQ direct attention and energy toward cross-cultural situations based on intrinsic interest (Deci and Ryan, 1985) and confidence in their cross-cultural effectiveness (Bandura, 2002). Behavioral CQ reflects the capability to exhibit appropriate verbal and non-verbal actions when interacting with people from different cultures. Thus, mental capabilities for cultural understanding and motivation need to be complemented with appropriate exhibitions of verbal and non-verbal actions, based on cultural values of specific settings (Hall, 1959). Those with high Behavioral CQ (Gudykunst et al., 1988) exhibit situationally appropriate behaviors based on their broad range of verbal (words and tone) and non-verbal (gestures and facial expressions) capabilities.

According to the authors (Earley and Ang, 2003), these four dimensions are qualitatively different facets of the overall capability to function and manage effectively in culturally diverse settings. CQ dimensions do not necessarily have to correlate with each other, but it is their combination that defines the overall CQ. Moreover, Ng and Earley (2006) say that CQ is a “culture-free” construct, not linked with academic intelligence. Defining CQ as a “culture-free” construct means that CQ is conceptualized as a set of competencies that can be increased over time and independently from the context (Earley and Peterson, 2004).

Regarding the development of the original version of the scale, here are some notes in detail.

To develop the Cultural Intelligence Scale (CQS), Earley and Ang (2003), analyzed literature on intelligence and intercultural competence and interviewed eight managers with extensive global professional experience. In the operationalization process they referred to different domains:

- educational and cognitive psychology (see O’Neil and Abedi, 1996) with regard to metacognition (awareness, planning, control and monitoring of mental, and learning process);
- Human Relations Areas (Murdock, 1987; Triandis, 1994);
- intrinsic satisfaction theory by Deci and Ryan (1985);
- Bandura’s theory of auto-efficacy in an intercultural context (2002);
- communication characteristics with regard to verbal and non-verbal flexibility (Hall, 1959; Gudykunst et al., 1988).

From authors’ analysis^1^, CFQ showed a good fit between the four-factors model and data collected in a sample of 576 university students from Singapore (**Table 1**).

**Table 1:** Overall model fit indices (Ang et al., 2007).

χ^2^ (gdl)	NNFI	CFI	SRMR	RMSEA	ρ
822.26 (164)	0.91	0.92	0.06	0.08	>0.70

Standardized factor loadings for items in the four scales (0.52–0.80) were significantly different from zero (*t*-values: 9.30–17.51, *p* < 0.05). The four factors had moderate inter-correlations (0.21–0.45) and acceptable variances (0.75–1.03). The corrected item-to-total correlations for each subscale (0.47–0.71) demonstrated strong relationships between items and their scales, supporting internal consistency.

Additionally, authors collected data from Singapore and the United States to verify their reliability across samples, time and countries with three cross-validation samples. Authors tested their hypotheses in three substantive studies.

### Cross-Validation of the Cultural Intelligence Scale (CQS) Across Samples

CFA on the first cross-validation sample (*N* = 447 undergraduates in Singapore) demonstrated good fit for the hypothesized four-factor model (**Table 2**).

**Table 2:** Cross-validation across sample fit indices (Ang et al., 2007).

χ^2^ (gdl)	NNFI	CFI	SRMR	RMSEA	ρ
381.28 (164)	0.96	0.96	0.04	0.05	>0.70

Standardized loadings (0.50–0.79) were significantly different from zero (*t*-values: 8.32–12.90, *p* < 0.05), with moderate correlations between factors (0.23–0.37) and acceptable variances (0.87–1.05). Corrected item-to-total correlations for each subscale (0.46–0.66) demonstrated strong relationships between items and their scales, supporting internal consistency.

### Generalizability of the Cultural Intelligence Scale (CQS) Across Time

A subset of respondents (*N* = 204) from the Singapore cross-validation sample completed the CQS again 4 months later. To examine T1–T2 longitudinal measurement invariance were used CFA and an augmented covariance matrix as input (rather than a multi-sample approach) to account for time-wise correlated errors. A 20-item was used by two-measurement occasion matrix and specified eight latent variables (four T1 CQ factors and four T2 CQ factors), with unique variances of identical items correlated across time. Results demonstrated acceptable fit (**Table 3**) suggesting that the four-factor model held across the two-time periods.

**Table 3:** Cross-validation across time fit indices (Ang et al., 2007).

χ^2^ (gdl)	NNFI	CFI	SRMR	RMSEA
981.18 (692)	0.94	0.95	0.06	0.04

The χ^2^ difference between Models A and B (factor loadings constrained to be invariant) failed to reach significance [Δχ^2^_(16_*_df_*_)_ = 22.79, *p* = ns], providing strong support for invariance in factor loadings across T1 and T2. The χ^2^ difference between Models B and C (item intercepts constrained to be invariant) also failed to reach significance [Δχ^2^_(14_*_df_*_)_ = 17.59, *p* = ns], providing support for item intercept invariance.

### Generalizability of the Cultural Intelligence Scale (CQS) Across Countries

The equivalence of the CQS in a US sample (*N* = 337) compared with the Singapore cross-validation sample (*N* = 447) was tested with sequential tests of model invariance. Model A (four factors with loadings freely estimated across samples) demonstrated good fit (**Table 4**), indicating equivalence in a number of factors.

**Table 4:** Cross-validation across countries fit indices (Ang et al., 2007).

χ^2^ (gdl)	NNFI	CFI	SRMR	RMSEA
723.23 (328)	0.96	0.97	0.05	0.05

The χ^2^ difference between Models A and B (four factors with loadings forced to be invariant) failed to reach significance [Δχ^2^_(16_*_df_*_)_ = 13.74, *p* = ns], providing strong support for invariance in factor loadings across settings. The χ^2^ difference between Models B and C (four factors with factor covariances forced to be invariant) failed to reach significance [Δχ^2^_(10_*_df_*_)_ = 17.96, *p* = ns], supporting invariance in factor covariances. In sum, multiple group tests of invariance demonstrated the same four factor structure holds across the two countries.

Moreover, Ang et al. (2007) tested three Hypothesis with three different kind of samples (undergraduates from United States and Singapore; international managers; foreign professionals and their supervisors). Hypothesis are reported below:

- Metacognitive CQ and Cognitive CQ as predictors of the Cultural Judgment and Decision Making (CJDM) effectiveness (undergraduates and international managers samples);
- Motivational CQ and Behavioral CQ as predictors of cultural adaptation (undergraduates foreign professionals and supervisors samples);
- Metacognitive CQ, Cognitive CQ, Motivational CQ, and Behavioral CQ relating positively to task performance.

As reported in the paper (Ang et al., 2007) the first Hypothesis was supported in both samples.

- Undergraduates–Metacognitive CQ (β = 0.21, *p* < 0.01/β = 0.15, *p* < 0.05) and Cognitive CQ (β = 0.16, *p* < 0.05/*β* = 0.13, *p* < 0.05) predicted CJDM;
- International managers–Metacognitive CQ (β = 0.30, *p* < 0.05) and Cognitive CQ (β = 0.37, *p* < 0.05) predicted CJDM.

Also, the second Hypothesis found confirmation with both samples.

- Undergraduates–Motivational CQ (β = 0.15, *p* < 0.05/β = 0.13, *p* < 0.05) and Behavioral CQ (β = 0.17, *p* < 0.05/β = 0.10, *p* < 0.05) predicted interactional adjustment; Motivational CQ (β = 0.16, *p* < 0.01/β = 0.12, *p* < 0.05) and Behavioral CQ (β = 0.13, *p* < 0.05/β = 0.09, *p* < 0.05) predicted wellbeing.
- Foreign professionals and supervisors–Motivational CQ and Behavioral CQ predicted interactional adjustment (β = 0.42, *p* < 0.01/β = 0.28, *p* < 0.05) and work adjustment (β = 0.41, *p* < 0.01/β = 0.35, *p* < 0.05). Motivational CQ and Behavioral CQ also predicted self-reported cultural adaptation: Motivational CQ and Interactional (β = 0.41, *p* < 0.001), work (β = 0.39, *p* < 0.001), and general adjustment (β = 0.33, *p* < 0.001) as well as well-being (β = 0.47, *p* < 0.001); Behavioral CQ and interactional adjustment (β = 0.27, *p* < 0.01), work adjustment (β = 0.19, *p* < 0.05), general adjustment (β = 0.26, *p* < 0.01), and wellbeing (β = 0.19, *p* < 0.05).

Finally, also the third Hypothesis was quite satisfactorily supported by data of both samples.

- International managers–Metacognitive CQ (β = 0.30, *p* < 0.05) and Behavioral CQ (β = 0.47, *p* < 0.001) predicted task performance. Cognitive CQ (β = 0.19, ns) and motivational CQ (β = -0.01, ns) did not significantly relate to task performance, and therefore H3b and H3c were not supported. CQ increased explained variance in CJDM by 22% (adjusted *R*^2^ = 0.21) and in task performance by 24% (adjusted *R*^2^ = 0.28), over and above sex, citizenship, cross-cultural experience, dyadic similarity, general mental ability, rhetorical sensitivity, and social desirability. Usefulness analysis shows variance explained by CQ (0.22–24) compared favorably with General Mental Ability (0.02–0.03), Rhetorical Sensitivity (0.01–0.05), and Social Desirability (0.07–0.09).
- Foreign professionals and supervisors–Metacognitive CQ (*β* = 0.47, *p* < 0.01) and Behavioral CQ (*β* = 0.31, *p* < 0.05) predicted supervisor rated task performance. Results did not support Cognitive CQ (*β* = 0.00, ns) or Motivational CQ (*β* = 0.26, ns) as predictors of task performance. CQ increased explained variance in supervisor rated task performance 36% (adjusted *R*^2^ = 0.29), interactional adjustment 28% (adjusted *R*^2^ = 0.18), and work adjustment 29% (adjusted *R*^2^ = 0.19). CQ also increased explained variance in self-rated interactional adjustment 26% (adjusted *R*^2^ = 0.26), work adjustment 19% (adjusted *R*^2^ = 0.16), general adjustment 20% (adjusted *R*^2^ = 0.30), and well-being 29% (adjusted *R*^2^ = 0.26). Usefulness analysis shows variance explained by CQ (0.19–36) compares favorably with variance explained by demographic characteristics of sex and cross-cultural experience (0.01–0.11).

According to these data, CQS seems to be a robust and reliable scale.

To summarize, these results support all elements up to now described, helping us to understand how the world of intercultural relationships is far more complex and comprehensive than we might believe. Generally, establishing and maintaining relationships seems obvious and trite, but relationships require strong awareness and motivation of the individual both in personal and professional life.

## Materials and Methods

### Objectives and Scope

The purpose of this study was to examine the validity of score interpretations of the Italian version of the CQS, a scale that seeks to measure individual ability to understand, act and manage effectively in culturally diverse settings.

In this study characteristics of internal consistency and correlations between factors will be described. Given that similar validated scales were not found for the Italian context, discriminant and predictive analysis will not be proposed.

### Sample

Participants were 755, 261 males (34.6%) and 485 females (64.2%), 9 participants did not answer this item. The clear majority of participants who responded (737) are Italian (90.8%)^2^. The age of participants who answered this question (786) is between 20 and 63 years and the subjects are distributed in a sufficiently balanced manner among the various age groups: 26.4% in the 20–32 age range; 26.9% in the 33–41 age range; 21.9% in the 42–48 age range; 24.9% in the 49–63 age range. The average age is 40.4 years (*SD* = 10.29). Among those who responded (717), the percentage of people working in companies slightly lower (28.5%) compared to those working in cooperatives (35.6%) and assisted living facilities (36.0%). With regard to the professional role, among those who responded (732), most belong to the category of care^3^ ASA/OSS (32.4%). These are followed by roles having decision making and planning functions (13.1%), with a socio-humanistic education (12.7%), and those with managerial functions (11.5%).

The different organizations that participated in the study were predominantly contacted in the Lombardy region (**Table 5**).

**Table 5:** Regional distribution of the organizations involved in the study.

Variables	Variables’ levels	Frequency %	Valid frequency %
Geographic region	Lombardy	85.0	91.7
	Trentino	2.0	2.1
	Veneto	0.4	0.4
	Emilia Romagna	1.1	1.1
	Friuli Venezia Giulia	3.4	3.7
	Piedmont	0.4	0.4
	Liguria	1.1	0.1
	Basilicata	1.1	0.1
	Sardinia	1.1	0.1
	Missing	7.3	/

The sampling criterion was one of convenience (no particular inclusion criteria were adopted except for the companies, which had to be involved in the manufacture of goods). Organizations were contacted with the help of two Federations, one for the manufacturers and one for the service-based companies.

### Measure

Cultural Intelligence was measured with the translated version of the CQS, which consists of 20 items referring to the four factors. Each subscale is composed of items that measure the construct in a direct way (the highest degree of agreement corresponds to the maximum degree of consensus with the detected perspective). All items are closed questions on a five-point Likert scale.

### Procedure

The Italian version of the scale presents the same theoretical structure with the four dimensions translated as: “Intelligenza Culturale Metacognitiva,” “Intelligenza Culturale Cognitiva,” “Intelligenza Culturale Motivazionale,” and “Intelligenza Culturale Comportamentale.” To guarantee the respect of the scale’s original meaning, a back-translation was realized by a psychologist, who is an expert of the construct, and by a native English language translator (**Table 6**).

**Table 6:** Italian translation of Cultural Intelligence Scale (CQS).

Factor	Item English version	Item Italian translation
Metacognitive CQ 4 items	- I am conscious of the cultural knowledge I use when interacting with people with different cultural backgrounds. - I adjust my cultural knowledge as I interact with people from a culture that is unfamiliar to me. - I am conscious of the cultural knowledge I apply to cross–cultural interactions. - I check the accuracy of my cultural knowledge as I interact with people from different cultures.	- Sono consapevole delle conoscenze culturali che utilizzo quando interagisco con persone con differenti background culturali. - Riesco ad adattare le conoscenze culturali quando interagisco con persone provenienti da una cultura che non-conosco. - Sono consapevole delle conoscenze culturali che applico in interazioni cross-culturali. - Verifico l’accuratezza delle mie conoscenze culturali quando interagisco con persone di differenti culture.
Cognitive CQ 6 items	- I know the legal and economic systems of other cultures. - I know the rules (e.g., vocabulary, grammar) of other languages. - I know the cultural values and religious beliefs of other cultures. - I know the marriage systems of other cultures. - I know the arts and crafts of other cultures. - I know the rules for expressing non-verbal behaviors in other cultures.	- Conosco i sistemi legali ed economici di altre culture. - Conosco le regole (es. vocabolario, grammatica) di altre lingue. - Conosco i valori culturali e le credenze religiose di altre culture. - Conosco i sistemi matrimoniali di altre culture. - Conosco arti e mestieri di altre culture. - Conosco le regole per esprimere i comportamenti non-verbali in altre culture.
Motivational CQ 5 items	- I enjoy interacting with people from different cultures. - I am confident that I can socialize with locals in a culture that is unfamiliar to me. - I am sure I can deal with the stresses of adjusting to a culture that is new to me. - I enjoy living in cultures that are unfamiliar to me. - I am confident that I can get accustomed to the shopping conditions in a different culture.	- Mi piace interagire con persone di differenti culture. - Sono fiducioso/a di poter socializzare con le persone di una cultura che non-conosco. - Sono sicuro/a di poter far fronte allo stress legato all’adattamento ad una nuova cultura. - Mi piace vivere in culture che non-conosco. - Sono sicuro/a di potermi abituare alle condizioni di compra-vendita in una cultura differente.
Behavioral CQ 5 items	- I change my verbal behavior (e.g., accent, tone) when a cross-cultural interaction requires it. I use pause and silence differently to suit different cross-cultural situations. - I vary the rate of my speaking when a cross-cultural situation requires it. - I change my non-verbal behavior when a cross-cultural situation requires it. - I alter my facial expressions when a cross-cultural interaction requires it.	- Cambio i miei atteggiamenti verbali (es. accento, tono) quando richiesto da un’interazione interculturale. - Uso pause e silenzi in modo diverso per adattarmi a differenti situazioni cross-culturali. - Modifico la velocità nel parlare quando richiesto da una situazione cross-culturale. - Modifico i miei comportamenti non-verbali quando richiesto da una situazione cross-culturale. - Modifico le mie espressioni facciali quando richiesto da un’interazione cross-culturale.

During the cross-cultural translation process, it is fundamental pay attention not just to linguistic aspects, but also to the sample’s psychological dimensions and the cultural characteristics of the context where data will be collected. Therefore, in order to verify the proper comprehension of the contents a pilot survey was planned with a variety of professionals with different levels of knowledge of the Italian language.

Data for the initial validation were collected by completing a paper version of the questionnaire (and, if required by the organization, in the presence of a researcher) or an online version on the Qualtrics platform (accessible through an anonymous dedicated link). The questionnaire consisted in closed questions and generally required a time of between 35 and 40 min to complete.

A total of 835 questionnaires were collected and were entered in a first database for preliminary analysis. Some were discarded because they did not meet the basic criteria (CQS not completed or many missing answers).

### Data Analysis

In this study we applied a Confirmatory Factor Analysis (CFA), which belongs to the Structural Equation Modeling (SEM) statistical methodology. CFA was calculated on EQS-6.3 Software (Byrne, 2006).

**FIGURE 1:**
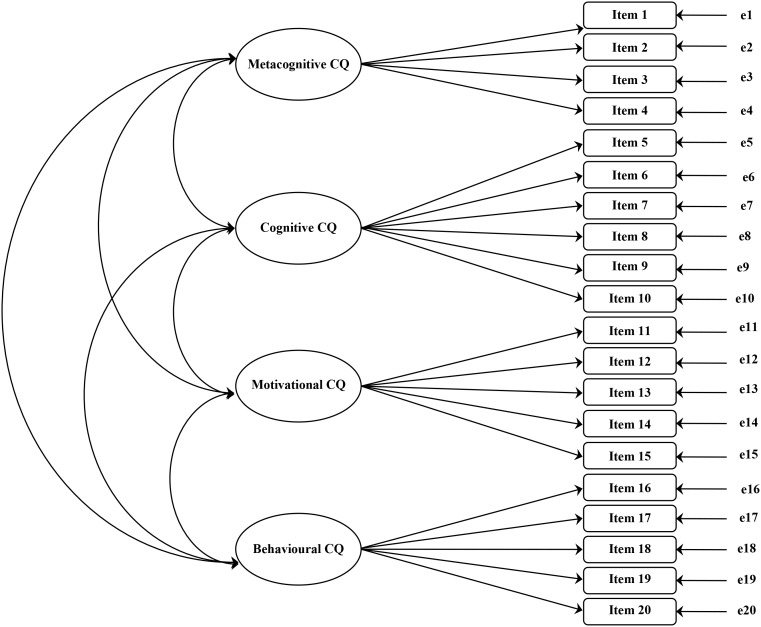
Four-factor Cultural Intelligence Scale (CQS) model tested in the study.

In their paper Ang et al. (2007) defined CQ as an aggregate multidimensional construct with four dimensions that are qualitatively different facets of the overall capability to function and manage effectively in culturally diverse settings. In light of the fact that authors proposed strong evidence in support of the structure of CQS, the first aim of this study was to identify the same factorial structure for the Italian sample. As illustrated in **Figure 1**, we tested a model with four different theoretical factors correlated with each other.

As regards the overall goodness of fit of the model, in this study different indices relating to the entire model and the individual parameters were considered: goodness-of-fit statistics [chi-square statistic (χ^2^), Non-Normed Fit Index (NNFI), Comparative Fit Index (CFI), Standardized Root Mean Square Residual (SRMR), Root Mean Square Error of Approximation RMSEA, and Raykov’s Reliability RHO (ρ)], Discriminant Reliability, Composite Reliability (or omega ω), and Factor Loadings (Bollen, 1989; Bentler, 1992; Corbetta, 1992; Browne and Cudeck, 1993; MacCallum et al., 1996; Raykov, 1998; Hu and Bentler, 1999; Hair et al., 2004; Byrne, 2006; Geldhof et al., 2014).

## Results

Analysis (**Table 7**) showed that the “CQS four-factor model” fit data satisfactorily.

**Table 7:** Fit indices–four-factor CQS model.

*N*	χ^2^ (gdl)	NNFI	CFI	SRMR	RMSEA	ρ
755	765.399^∗^ (164)	0.92	0.93	0.05	0.07^∗∗^	0.95

*^∗^The probability value for the chi-square statistic is 0.00000. However, χ^2^ is sensitive to the sample size; with a big sample size it is highly probable to have a p < 0.05, even if the model fits the data (Corbetta, 1992). ^∗∗^MacCallum et al. (1996) suggest that values from 0.08 to 0.10 indicate a mediocre but still acceptable fit.*

Given that the overall model’s indices were good, we decided to verify if it could be possible to increase its adherence with empirical data. Factor loading, suggestion for change and correlations were verified. All items (**Table 8**) showed optimal saturation level for their own factor (range between 0.628 and 0.880). For this reason, no changes were made.

**Table 8:** Items’ standardized solutions-four-factor CQS model.

Factor	Item	Standardized solution
Metacognitive CQ	1	0.752
	2	0.837
	3	0.856
	4	0.710
Cognitive CQ	5	0.734
	6	0.628
	7	0.770
	8	0.817
	9	0.806
	10	0.717
Motivational CQ	11	0.830
	12	0.817
	13	0.784
	14	0.803
	15	0.697
Behavioral CQ	16	0.724
	17	0.825
	18	0.843
	19	0.880
	20	0.800

As reported in **Table 9**, the Composite Reliability (or omega ω) was considered satisfying for each of our four factors (range between 0.80 and 0.84).

**Table 9:** Composite reliability of CQS’ four factors.

Factors	ω
Metacognitive CQ	0.80
Cognitive CQ	0.84
Motivational CQ	0.83
Behavioral CQ	0.83

Consistently with authors’ statements, CQS’ four dimensions showed moderate correlation with each other (**Table 10**).

**Table 10:** Correlations among CQS factors-four-factor CQS model.

Factors	Correlations among independent variables values
Metacognitive CQ–cognitive CQ	0.62
Metacognitive CQ–motivational CQ	0.59
Metacognitive CQ–behavioral CQ	0.42
Cognitive CQ–motivational CQ	0.54
Cognitive CQ–behavioral CQ	0.38
Motivational CQ–behavioral CQ	0.49

In summary, as proposed in the original scale, our final version is composed of 20 items and 4 different theoretical dimensions (Metacognitive, Cognitive, Motivational, and Behavioral) that correlate with each other.

In this study, internal consistency has obtained very good indices that seem to imply an optimal reliability for the Italian context.

We can say that all these results are in line with all the results obtained by Ang et al. (2007) during cross-validation across sample, time, and countries.

As affirmed earlier there are no other CQSs for the Italian context so it hasn’t been possible take into account discriminant and predictive analysis.

## Discussion

Cultural Intelligence Scale is a scale that seeks to measure an individual’s ability to understand, act and manage effectively in culturally diverse settings. CFA results supported CQS’s satisfying psychometric characteristic. In line with the findings of Ang et al. (2007), the results of this contribution seem to provide empirical support for CQS’s reliability, confirming the existence of four specific dimensions of cultural intelligence: Metacognition, Cognition, Motivational, and Behavioral.

These results can be seen as a support for the use of this scale in different domains (from research to Diversity Management). Therefore, being able to deal with differences, is not only an opportunity for personal and professional growth, but also a resource for organizations themselves. In an organization, focusing on professionals’ CQS level could be helpful to design and implement specific HR policies.

## Conclusion

This study has its limits. While the purpose was to offer a contribution validating CQS for the Italian context, we did not achieve a geographical representativeness applicable to the national working population. Future research could try to move beyond this limit through more extensive data collection in order to achieve a better geographical and socio-cultural representativeness. Furthermore, in order to further solidify these promising initial findings, future studies could test (compared with other instruments) the stability and predictive potential of the scale in the Italian context. In fact, as already underlined by Ang et al. (2007) it would be interesting to understand how CQS interacts with variables linked not only to individual factors (such as self-monitoring, need for cognition, need for closure, self-efficacy, ethnocentrism, self-construal, and social identity) but also with factors related to group and organizational dimensions (conflict management, sharing of the work object, group and organizational creativity, collaboration). Moreover, it could be interesting to combine CQS’s items with a more projective tool in order to explore more complex dynamics. For example, thanks to the format’s cultural sensibility, vignettes are particularly suitable for assessing perceptions of Diversity and Cultural Intelligence.

Despite these limits, this study could be considered a first contribution to bridge the gap of self-report measure concerning cultural intelligence in the Italian context and, more broadly, to develop a deeper comprehension of Diversity and Diversity Management in the organizational context.

## Ethics Statement

This study was carried out in accordance with the recommendations of “Commissione Etica per la Ricerca in Psicologia, Dipartimento di Psicologia-Università Cattolica del Sacro Cuore” with written informed consent from all subjects. All subjects gave written informed consent in accordance with the Italian Legislative Decree 30 June 2003 no. 196. The written informed consent is reported below: “I agree to the proposal to participate in the research study. My agreement is an expression of a free decision, not influenced by promises of economic benefits or otherwise, nor from obligations to the principal investigator of the study. I am aware of being free to withdraw from the study at any time I want. Moreover, I am aware that I’m not supposed to give any reasons for my decision to withdraw from the study. I was given the opportunity to ask questions about the aims and methods of the study and my rights as a participant in the research. I understood all the information and explanations that I have been given and I had enough time to consider my participation in this study. According to the Italian Legislative Decree 30 June 2003 no. 196, I agree that my personal data will be used for specific research purposes within the limits and in the manner please explain in the information document.” The protocol was approved by the “Commissione Etico per la Ricerca in Psicologia”.

## Author Contributions

All authors listed have made a substantial, direct and intellectual contribution to the work, and approved it for publication.

## Conflict of Interest Statement

The authors declare that the research was conducted in the absence of any commercial or financial relationships that could be construed as a potential conflict of interest.

## Footnotes

1 Here is proposed an extract with the results from the paper by Ang et al. (2007).

2 We decided to include also the non-Italian respondents because all of them were living in Italy for at least 10 years, have a job and speak Italian fluently. For all these reasons we thought that it could have been important include them in the sample. Moreover, they were representative of the professional composition of the organizations involved.

3 These professional categories—typical of facilities offering assisted living –provide people with care services such as personal hygiene, feeding.
